# Supplementary material for: Vitamin C Regulates the Profibrotic Activity of Fibroblasts in In Vitro Replica Settings of Myocardial Infarction
Source: Int J Mol Sci. 2023 May 6;24(9):8379. doi: 10.3390/ijms24098379 (PMC10179686; doi:10.3390/ijms24098379)
Supplement: Supplementary file 1 [file ijms-24-08379-s001.zip › ijms-2303686-supplementary.pdf]

Supplementary Material

S-1

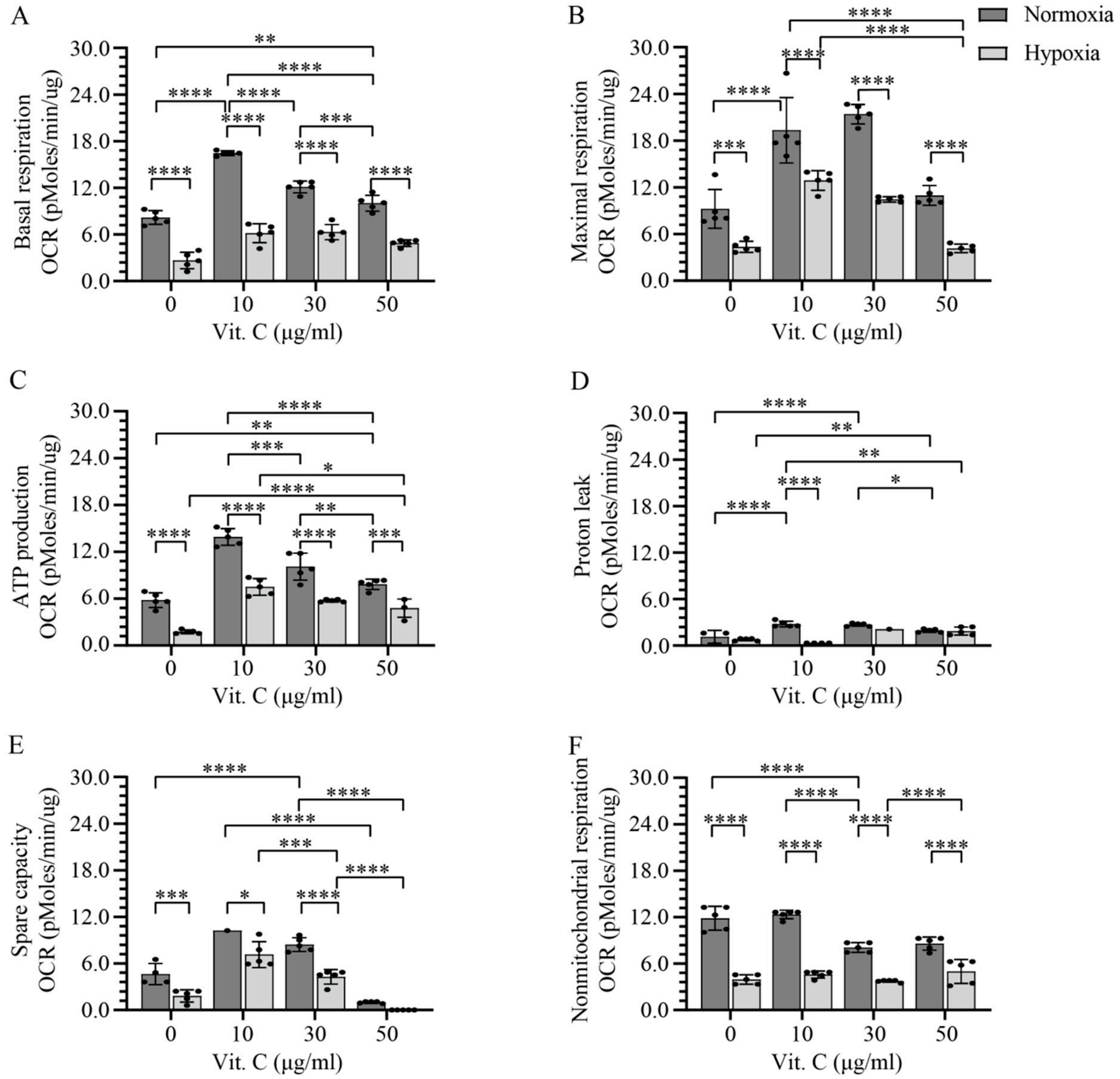

Figure-S1. A maximum threshold level of 10 µg/ml of vitamin C shows a positive effect on the metabolic profile of mitochondria at the normal and minimal O_2 availability. The increased basal and maximal respiration (**A, B**) which associates to the elevation of ATP production (**C**) and H^+ leak levels (**D**). Increased vitamin C concentration (50 µg/ml) reduced significantly the spare capacity of mitochondria (**E**). Only in normal O_2 availability conditions increased vitamin C concentrations (30 µg/ml and 50 µg/ml) lead to reduced nonmitochondrial respiration, whereas these values are similarly to those of control and are independent on vitamin C concentration, under low O_2 availability (**F**). Data are normalized to total protein. Error bars indicate standard error of the mean; $^{*}p \le 0.05$, $^{**}p \le 0.01$, $^{***}p \le 0.001$, $^{****}p \le 0.001$, n = 3 experiments per condition. (Vit. C = vitamin C)

S-2

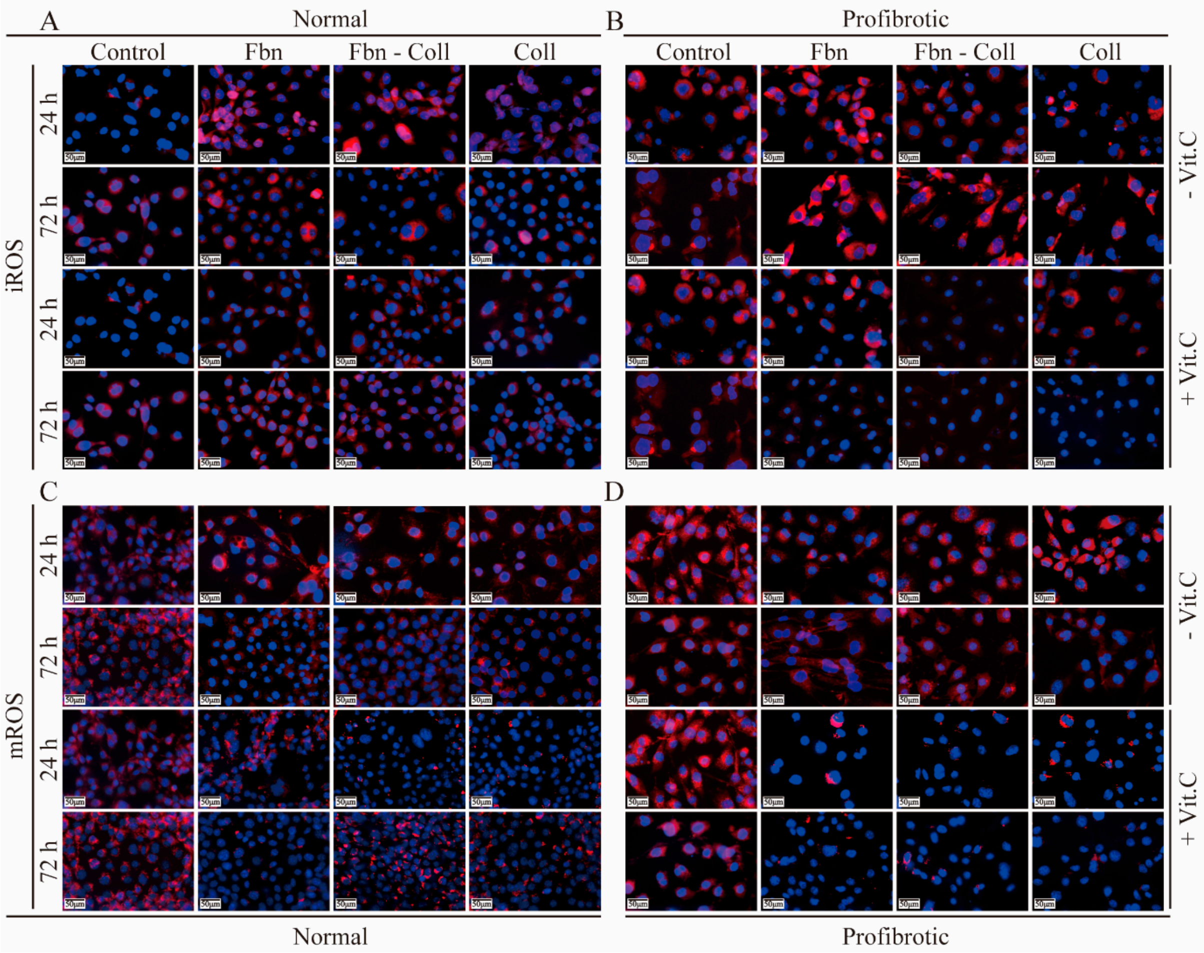

Figure-S2. Differential reduction of iROS vs. mROS by vitamin C potentiated by normoxia and profibrotic conditions. Fluorescent images of intracellular ROS (iROS) detection by DHE (bright fluorescent red)-DAPI (blue) in (**A**) normoxia and (**B**) profibrotic settings, respectively. Complementary, mitochondrial ROS (mROS) was detected via mitoSOX – DAPI co-staining of fibroblasts (10 000 cells/ well) in normoxia (**C**) and profibrotic (**D**), respectively. n = 3 experiments per condition. Scale indicates 50 µm. (Vit. C = vitamin C, Fbn = Fibronectin, Coll = Collagen)

S-3

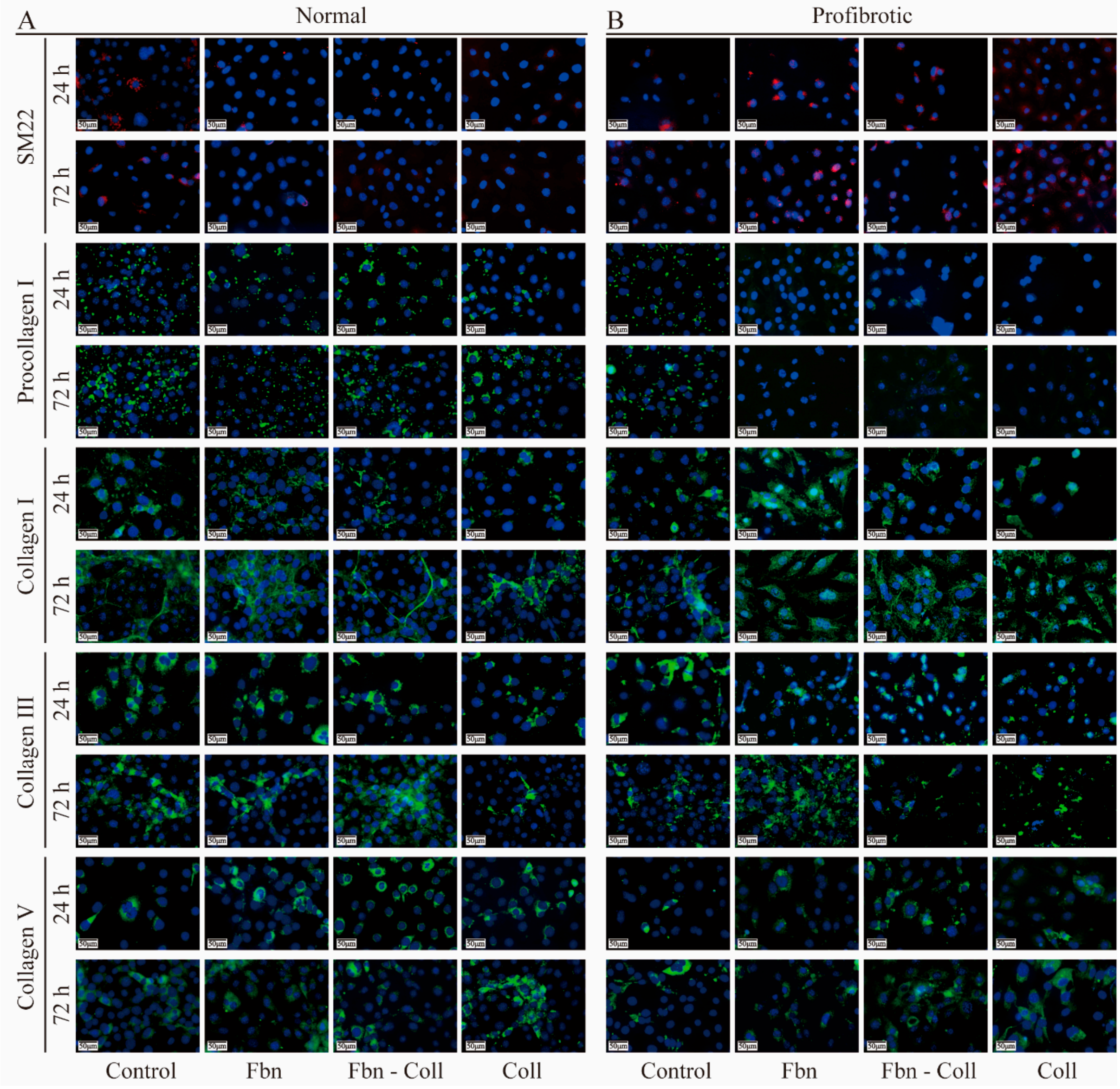

Figure-S3. Profibrotic settings mediate the expression of SM22 expression associated to distinct type I, III, and V collagen synthesis and distribution. The cultivated primary fibroblasts were differentiated to myofibroblasts when exposed to in profibrotic conditions for 24 hours and 72 hours. Immunofluorescent images illustrate the colocalization of DAPI (blue)-SM22 (red) of primary fibroblasts incubated for 24 hours and 72 hours, in normoxia (A- top, left). The quality of fibroblasts is largely affected under profibrotic settings compared to those cultured in normal conditions. Under profibrotic settings, particularly on fibronectin SM22 is equally more expressed at 24 hours and 72 hours. (B-top, right). Fluorescent intensities of type I procollagen increase at 72 hours, in normoxia (A-middle, top, left) whereas in profibrotic its intensity vanishes in time (B-middle, top, left). Fluorescent intensity of type I collagen increases in both normoxia (A- **middle, left**) and profibrotic (**B- middle, right**) at 72 hours. Similarly, the fluorescence of type III collagen in normoxia (**A- middle, bottom, left**) seems to be smaller than that in profibrotic (**B- middle, bottom, right**) at 72 hours whereas the fluorescent intensity of type V collagen increases with the incubation time, in normoxia (**A- bottom, left**) and profibrotic (**B- bottom, right**). n = 3 experiments per condition. Scale indicates 50 µm. (Fbn = Fibronectin, Coll = Collagen)

S-4

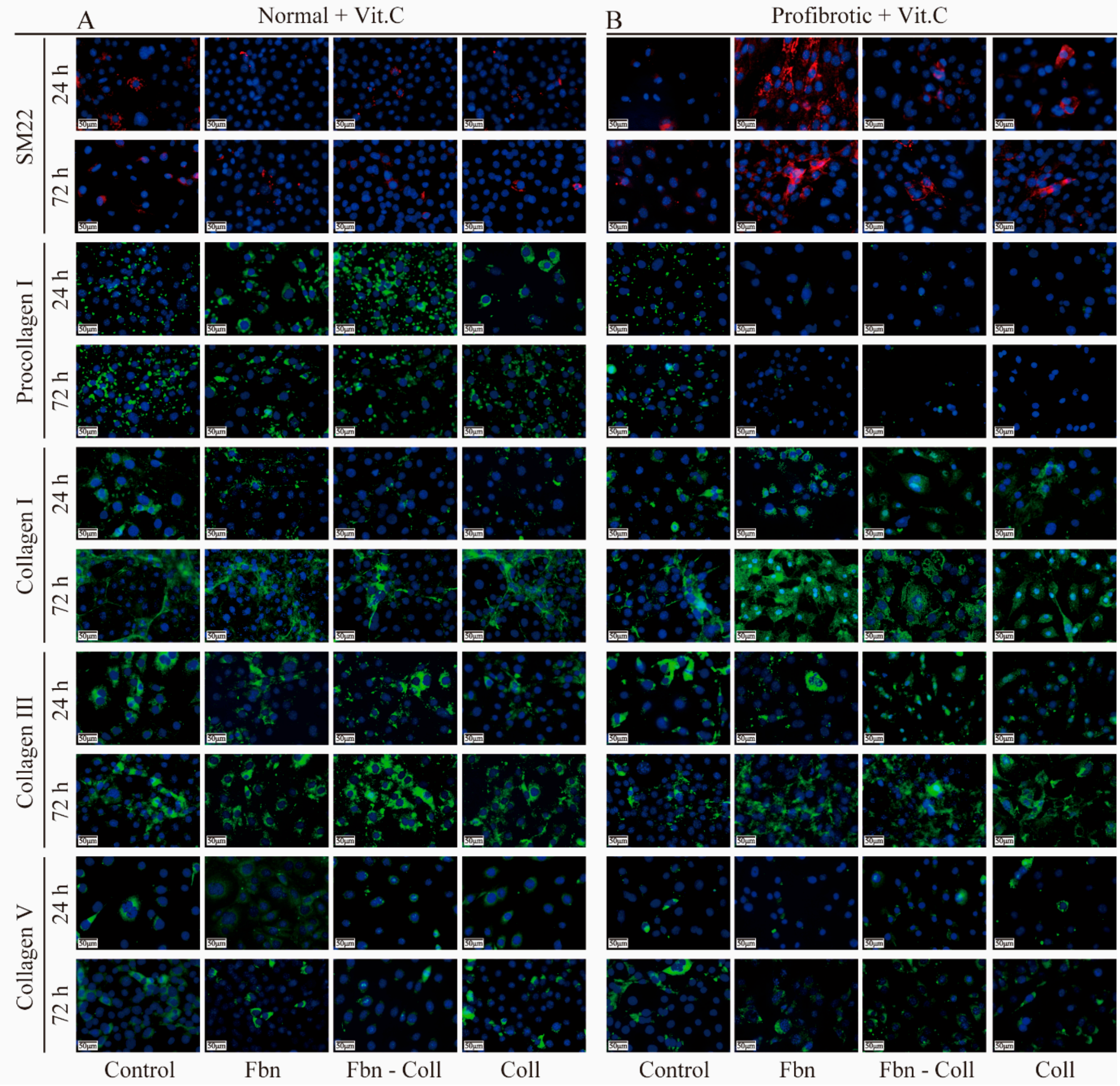

Figure-S4. The combined effects of vitamin C and profibrotic settings mediate the expression of SM22 expression associated to distinct type I, III, and V collagen synthesis and distribution. Fluorescent intensity variation in time of DAPI (blue)-SM22 (red) of primary fibroblasts under vitamin C supplementation in normoxia (**A- top, left**). Qualitatively, under the combined effect of vitamin C and profibrotic settings, SM22 expression is highly upregulated on fibronectin as an indication of fibroblasts activity modification. (**B-top, right**). Qualitatively, vitamin C has barely no influence on fluorescent intensities of type I procollagen in normoxia, at both 24 hours and 72 hours (**A-middle, top, left**) but, it vanishes their intensities in profibrotic (**B-middle, top, left)**. Independent of scaffold composition, vitamin C increases the fluorescent intensity of type I collagen at 72 hours, in normoxia (**A- middle, left**) and it enhances even more in profibrotic (**B- middle, right**). Under the influence of vitamin C, the fluorescent intensity of type III collagen becomes more intense in time for co-assembled systems than other once, in normoxia (**A- middle, bottom, left**) whereas in profibrotic the pronounced fluorescent intensity is evidenced in all scaffold systems (**B- middle, bottom, right**) at 72 hours. Although the fluorescent intensity of type V collagen in normoxia exhibits a minimum value at 24 hours, particularly the collagen scaffold has a positive feedback on type V collagen at 72 hours (**A- bottom, left**) whereas in profibrotic (**B- bottom, right**) its expression increases at 72 hours, independent of coating compositions. n = 3 experiments per condition. Scale indicates 50 µm. (Vit. C = vitamin C, Fbn = Fibronectin, Coll = Collagen)
